# Analysis of nAChR Autoantibodies Against Extracellular Epitopes in MG Patients

**DOI:** 10.3389/fneur.2022.858998

**Published:** 2022-03-28

**Authors:** Maria Michail, Vasiliki Zouvelou, Maria Belimezi, Anna Haroniti, Marios Zouridakis, Paraskevi Zisimopoulou

**Affiliations:** ^1^Laboratory of Molecular Neurobiology and Immunology, Hellenic Pasteur Institute, Athens, Greece; ^2^Department of Biology, National and Kapodistrian University of Athens, Athens, Greece; ^3^Department of Neurology, Eginition Hospital, National and Kapodistrian University of Athens, Athens, Greece; ^4^Diagnostic Department, Hellenic Pasteur Institute, Athens, Greece

**Keywords:** myasthenia, autoantibodies, anti-nAChR antibodies, cell-based assay, diagnosis of myasthenia, immunoadsorption

## Abstract

Myasthenia gravis (MG) is an autoimmune disorder caused by autoantibodies targeting components of the postsynaptic membrane of the neuromuscular junction (NMJ), leading to neuromuscular transmission deficiency. In the vast majority of patients, these autoantibodies target the nicotinic acetylcholine receptor (nAChR), a heteropentameric ion channel anchored to the postsynaptic membrane of the NMJ. Autoantibodies in patients with MG may target all the subunits of the receptor at both their extracellular and intracellular regions. Here, we combine immunoadsorption with a cell-based assay to examine the specificity of the patients' autoantibodies against the extracellular part of the nAChR. Our results reveal that these autoantibodies can be divided into distinct groups, based on their target, with probably different impacts on disease severity. Although our findings are based on a small sample group of patients, they strongly support that additional analysis of the specificity of the autoantibodies of patients with MG could serve as a valuable tool for the clinicians' decision on the treatment strategy to be followed.

## Introduction

Myasthenia gravis (MG) is a well-characterized autoimmune disorder caused by autoantibodies (autoAbs) targeting molecules of the neuromuscular junction (NMJ). In MG, the signal transduction caused by the neurotransmitter acetylcholine is impaired and muscle weakness and fatigability occur (1–4).

To date, various MG-specific autoAbs have been identified. One case is the autoAbs against the muscle nicotinic acetylcholine receptor (nAChR) (5), which act according to one of the following three pathogenic mechanisms: (a) activation of the complement at the NMJ, which causes destruction of the typical folds in the sarcolemma, (b) antigenic modulation, which leads to internalization and degradation of the surface nAChR, or (c) blocking of the acetylcholine binding and consequently of the channel opening (6). AutoAbs against the muscle-specific kinase (MuSK) (7) and low-density lipoprotein receptor-related protein 4 (LRP4) (8–10) block the interactions of MuSK and LRP4 and affect the maintenance of the NMJ (6, 11). In addition, other autoAbs with unknown pathogenicity directed against agrin, cortactin, titin, and ryanodine receptor have also been detected in patients' sera with MG (12–16).

The nAChR is a ligand-gated ion channel anchored to the NMJ (17). In humans, two subtypes of the muscle nAChR have been identified, the fetal and the adult subtype. Both the subtypes are heteropentamers composed of 4 subunits forming pentameric assemblies with a stoichiometry of 2α1: β1: ε: δ (adult subtype) or 2α1: β1: γ: δ (fetal subtype) (18–20). Each subunit consists of a ~210 amino acid extracellular domain (ECD), bearing the epitopes for potential pathogenic autoAbs (21, 22). Although the α1 subunit hosts the main immunogenic region, patients with MG also harbor autoAbs against the non-α1 subunit-ECDs (22–25). AutoAbs against the α1 subunit of the nAChR are characterized as more pathogenic than those against the β1 subunit (26). Furthermore, autoAbs against the γ subunit trigger arthrogryposis in newborns and recognize the fetal subtype of the nAChR on the extraocular muscle in adults (27–30). Thus, the subunit specificity of the anti-nAChR autoAbs seems to influence disease severity.

Currently, the gold standard technique for anti-nAChR autoAbs detection and quantification is a radioimmunoprecipitation assay (RIPA), performed with a mixture of solubilized fetal and adult human nAChR bound to the [^125^I]-labeled antagonist α-bungarotoxin. RIPA is a reliable technique that provides an accurate estimation of the anti-nAChR autoAbs titer (5, 31). The anti-nAChR autoAbs titer does not correlate with disease severity when patients are compared, although fluctuations in the anti-nAChR autoAbs concentration in an individual patient have been reported to correlate with the severity of muscle weakness and to predict exacerbations. Thus, repeated testing for autoAbs can influence therapeutic decisions (2). Other techniques with good sensitivity and specificity for the detection of the anti-nAChR autoAbs, namely, ELISA, luciferase and fluorescence immunoprecipitation assays, exist. However, these assays have not been widely adopted in clinical practice (2, 32–34). Recently, cell-based assays (CBAs) for the detection of anti-nAChR autoAbs have been developed (35). In brief, CBA utilizes either transiently or stably co-transfected cells with plasmids encoding the five subunits of the nAChR and rapsyn. This co-transfection results in overexpression of the native nAChR on the cell membrane, mimicking the tightly clustered nAChRs on the NMJ. Thus, in addition to other techniques, CBA allows the detection of conformational dependent anti-nAChR autoAbs that recognize discontinuous epitopes and clustered nAChRs (36–41). It has been reported that 16–66% of seronegative patients with MG have autoAbs against the clustered nAChR, detected by CBA (42–46).

Here, we studied the specificity of the anti-nAChR autoAbs in sera, derived from a group of 20 anti-nAChR positive patients with MG at different time points. First, we investigated how many of these patients possess autoAbs against extracellular parts of the nAChR by CBA. We were surprised to find that 7 out of the 20 patients with MG were CBA negative (CBA–), which suggests that they mainly have autoAbs against intracellular parts of the receptor, since these patients were RIPA positive against the native nAChR. Then, only for the CBA positive sera (CBA+), we tested by immunoadsorption the subunit specificity of the autoAbs. Following the immunoadsorption of autoAbs against specific subunit-ECDs, we tested the remaining autoAbs by: (a) RIPA to quantify the percentage of the unbound autoAbs and (b) CBA to test if all the autoAbs against extracellular parts of the nAChR were depleted. Based on our findings, we could divide the tested patients with MG into four groups, according to the target of their autoAbs, which possibly reflects differences in their clinical phenotype.

## Materials and Methods

### Patients

Sera from patients with nAChR-MG, confirmed by RIPA, were provided by the diagnostic department of the Hellenic Pasteur Institute (HPI). The sera samples used were collected from at least two different time points for most of these patients. In total, 55 sera were collected from 20 patients with MG (Table 1). Clinical data from 9 patients are available and given in Table 1.

**Table 1:** Results of the tested sera.

	**Date**	**Age**	**Onset**	**MGFA**	**anti-nAChR (nM)**	**Live CBA**	**anti-α1 (%)**	**anti-β1 (%)**	**anti-γ (%)**
**Live CBA negative**									
P1	2017	n.a.	n.a.	n.a.	820	N	N	N	N
	2021	n.a.	n.a.	n.a.	496	A	N	N	N
P2	2011	n.a.	n.a.	n.a.	275	N	N	N	N
	2013	n.a.	n.a.	n.a.	450	A	N	N	N
	2018	n.a.	n.a.	n.a.	256	A	N	N	N
	2019	n.a.	n.a.	n.a.	480	A	N	N	N
P3	2017	n.a.	n.a.	n.a.	97	A	N	N	N
	2019	n.a.	n.a.	n.a.	39	A	N	N	N
	2020	n.a.	n.a.	n.a.	28	A	N	N	N
P4	2017	n.a.	n.a.	n.a.	65	N			
	2018	n.a.	n.a.	n.a.	46	A			
P5	2011a	28	Early	IIA	272	N			
	2011b	28		IIA	277	N			
	2018	35		PR	130	N			
P6	2010	33	Early	I	341	N			
	2019	42		I	310	N			
P7	1999	51	Early	I	144	N			
	2009	61		I	145	N			
**Anti-α1 autoAbs**									
P8	2018	79	Late	I	8.8	P	57.84 (±6.07)	N	N
	2019	80		V	36	P	58.38 (±8.83)	N	N
P9	2017	n.a.	n.a.	n.a.	32	P	38.38 (±4.08)	N	N
	2018	n.a.	n.a.	n.a.	36	P	27.55 (±1.82)	N	N
P10	2016	n.a.	n.a.	n.a.	82	P	87.43 (±0.08)	N	N
	2017	n.a.	n.a.	n.a.	40	P	88.06 (±2.16)	N	N
	2020	n.a.	n.a.	n.a.	33	P	88.00 (±1.06)	N	N
P11	2016	n.a.	n.a.	n.a.	16	P	90.70 (±1.65)	N	N
	2017	n.a.	n.a.	n.a.	9	P	93.20 (±1.32)	N	N
	2020	n.a.	n.a.	n.a.	18.7	P	85.47 (±0.31)	N	N
P12	2011	n.a.	n.a.	n.a.	165	P	90.69 (±0.67)	N	N
	2012	n.a.	n.a.	n.a.	77	P	89.61 (±0.12)	N	N
	2019	n.a.	n.a.	n.a.	48	P	92.59 (±0.13)	N	N
**Non anti-α1 autoAbs**									
P13	2016	73	Late	I	165	P	N	N	65.33 (±2.48)
	2018	75		I	160	P	N	N	67.50 (±3.28)
P14	2016	n.a.	n.a.	n.a.	26	P	N	76.45 (±0.64)	N
	2020a	n.a.	n.a.	n.a.	153	P	N	39.69 (±1.43)	N
	2020b	n.a.	n.a.	n.a.	89	P	N	41.52 (±4.58)	N
	2021	n.a.	n.a.	n.a.	43	P	N	51.90 (±1.82)	N
P15	2013	n.a.	n.a.	n.a.	420	P	N	60.83 (±2.21)	N
	2016	n.a.	n.a.	n.a.	193	P	N	52.08 (±2.33)	N
	2020	n.a.	n.a.	n.a.	208	P	N	57.17 (±1.53)	N
**Anti-α1 and non anti-α1 autoAbs**									
P16	2017	36	Early	IIA	98	P	27.97 (±3.97)	N	16.15 (±0.83)
	2020	39		IIA	246	P	25.07 (±4.51)	N	15.83 (±7.85)
P17	2007	61	Late	IVB	25.4	P	18.21 (±5.59)	20.01 (±0.73)	34.50 (±4.48)
	2017a	71		IIA	3.5	P	17.51 (±4.32)	14.03 (±3.18)	63.50 (±4.47)
	2017b	71		IIA	2.5	P	16.11 (±1.73)	18.51 (±5.06)	53.51 (±2.13)
	2020	74		IIA	22	P	N	N	38.53 (±5.43)
P18	2011a	n.a.	n.a.	n.a.	65	P	37.37 (±5.16)	N	36.51 (±0.86)
	2011b	n.a.	n.a.	n.a.	14	P	38.38 (±1.54)	N	34.93 (±0.95)
	2017	n.a.	n.a.	n.a.	7.6	P	N	N	50.71 (±0.99)
	2019	n.a.	n.a.	n.a.	12.7	P	59.66 (±1.69)	N	18.26 (±1.36)
P19	2007	48	Early	IVB	8.2	P	56.85 (±6.70)	N	N
	2009	50		IIIB	5	P	N	50.94 (±0.85)	N
P20	2015	34	Early	IVB	11	P	17.51 (±5.96)	N	25.15 (±3.74)
	2018	37		IIB	9.6	P	26.51 (±6.01)	N	35.01 (±7.07)
	2020	39		IIB	198	P	N	44.12 (±4.94)	33.21 (±3.17)

*Patients with myasthenia gravis (MG) are grouped by autoAbs specificity. The year of the sample collection, the age of the patients, and the time of disease onset are listed. The distribution and severity of myasthenic weakness were classified according to the MG Foundation of America (MGFA) grading system. The titer of the anti-nAChR autoAbs is given as estimated by RIPA. All the sera were tested for the presence of anti-nAChR autoAbs targeting the extracellular part of the receptor by CBA. The sera were also tested for the presence of the autoAbs against each ECD of the five subunits of the receptor and the percentage of immunoadsorption presented here was estimated as described in “Materials and Methods” section. The average percentage of immunoadsorption from three experiments is presented. In parenthesis, the numbers refer to the ±SD of the immunoadsorption percentage between the different experiments (there was no depletion of autoAbs after the treatment with δ and ε ECD sepharose beads and thus these are not shown in the table)*.

*N, negative; A, ambiguous; P, positive; n.a., not available; PR, pharmacology remission*.

### Statement of Ethics

The studies involving human participants were reviewed and approved by HPI Ethics Committee. The patients/participants provided their written informed consent to participate in this study.

### Immobilization of Purified Recombinant Proteins on CNBr-Sepharose Beads

The expressed, in yeast *Pichia pastoris*, ECDs of the human α1, β1, γ, δ, and ε nAChR subunits (47, 48) were immobilized on cyanogen bromide (CNBr)-sepharose beads, after their enzymatic deglycosylation and purification, as described previously (24, 49). In brief, 0.1 mg of ECD and 0.9 mg of bovine serum albumin (BSA) (used as a carrier) were immobilized on 0.1 g of CNBr-activated sepharose beads according to the manufacturer's protocol (GE Healthcare). Following the immobilization, the ECD-carrying beads were diluted in 12 ml phosphate-buffered saline (PBS) containing 0.02% NaN_3_. As a control, BSA (1 mg) was immobilized on CNBr-activated sepharose beads.

### Immunoadsorption

A total of 125 fmoles of anti-nAChR autoAbs, diluted in PBS/0.2% BSA (total volume: 40 μl), were incubated with 120 μl of sepharose-ECD or sepharose-BSA suspension, for 2 h at room temperature (RT). After centrifugation, supernatants from the immunoadsorption columns containing the unbound anti-nAChR autoAbs were tested by RIPA and CBA.

### Radioimmunoprecipitation Assay

For the quantification of the unbound anti-nAChR autoAbs, the autoAb RIPA kit (RSR, UK), containing [^125^I]-α-bungarotoxin-labeled human fetal and adult muscle nAChR preparations, was used, according to the manufacturer's instructions. From the 160 μl immunoadsorption mix, duplicates of 30 μl samples (containing ~25 fmoles in case of no depletion) were added to the reaction. The percentage of immunoadsorption was estimated using the equation: 100 × {[ΔcpmBSA] – [ΔcpmECD]}/[ΔcpmBSA], where Δcpm is the cpm of [^125^I]-α-bungarotoxin-labeled nAChR (provided in the RSR kit) precipitated by the serum minus that precipitated by a control normal human serum and ΔcpmBSA and ΔcpmECD are the corresponding Δcpm values for samples incubated with immobilized BSA or nAChR-ECD, respectively.

### Cell-Based Assay

The CBA was performed as described by Leite et al. (35). Briefly, HEK293T cells were transiently co-transfected with the plasmids encoding for human α1, β1, γ, δ, and ε nAChR subunits and for rapsyn in a ratio of 2:1:1:1:1:1, respectively. Transfection was performed with polyethylenimine (Polyplus). After 48 h, the transfected cells were incubated with serum (20 fmoles of anti-nAChR autoAbs) or supernatant from the immunoadsorption mixture (30 μl containing ~20 fmoles if no depletion occurred) for 1 h at RT. Afterwards, cells were fixed in 10% formalin solution (Sigma-Aldrich) for 10 min at RT. Patients' anti-nAChR autoAbs were detected after incubation of the cells for 1 h at RT with Alexa Fluor-555 conjugated anti-human IgG Ab (Life Technologies, Invitrogen) in 1:750 dilution. The presence of nAChR on the cell surface was verified by staining with Alexa Fluor-488 labeled α-bungarotoxin (Life Technologies, Invitrogen) in 1:1,000 dilution. Cells were examined under an Olympus IX51 fluorescence microscope by 2 observers.

## Results

### Detection of AutoAbs Against the Extracellular Parts of the nAChR

**Figure 1:**
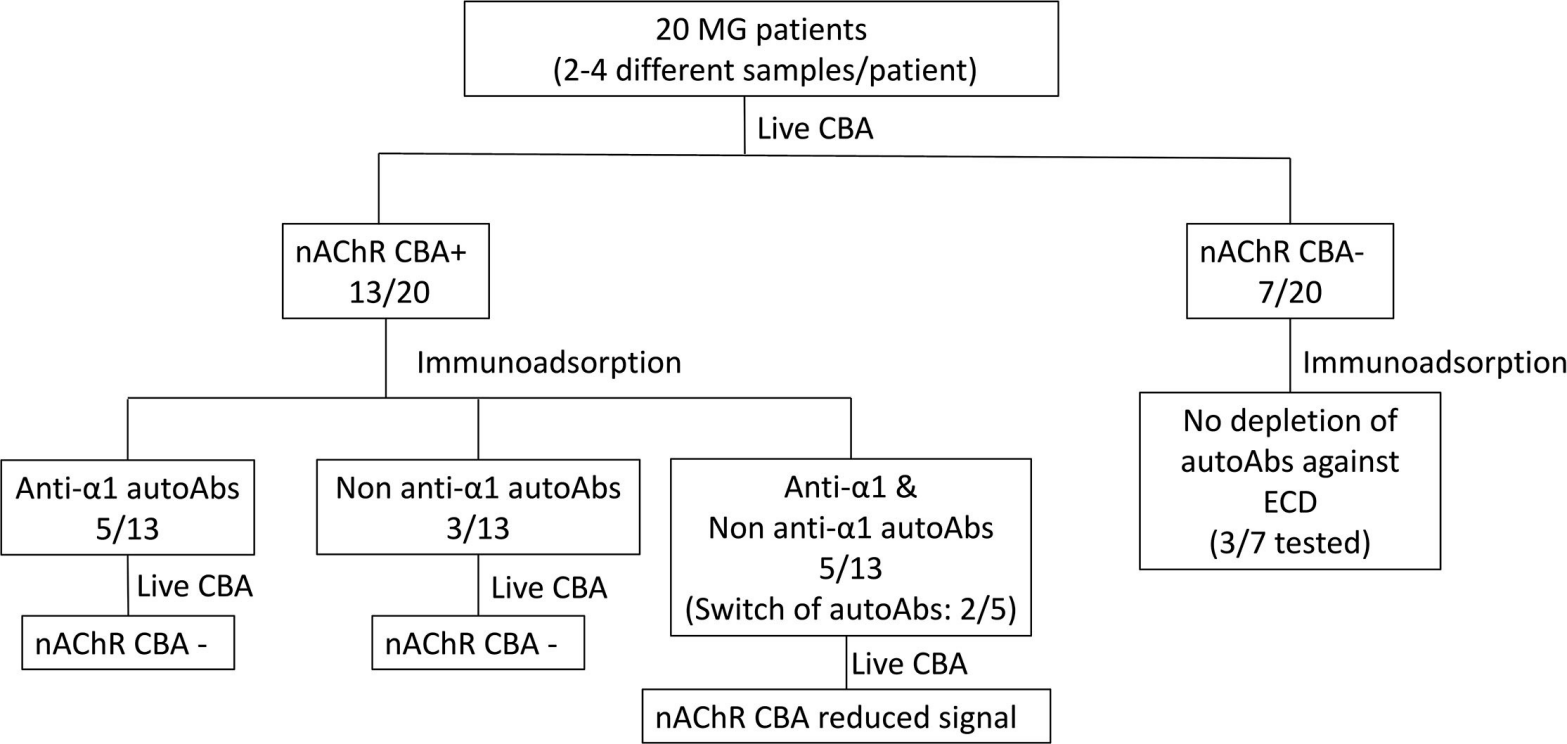
Specificity of the autoAbs derived from patients with myasthenia gravis (MG). Overview of the results.

We used sera from patients who were tested positive for the presence of anti-nAChR autoAbs at the diagnostic department of the HPI. We chose 20 patients with MG, from whom a recent and at least one previous serum sample were available (55 sera in total). In addition, clinical data for 9 out of the 20 patients were available (Table 1). The anti-nAChR autoAbs titer was estimated in all the sera by RIPA (Table 1), which detects autoAbs targeting both the extra- and intracellular parts of the nAChR, since solubilized intact nAChRs are used (5, 31). We tested samples containing 20 fmoles of anti-nAChR autoAbs from all sera by CBA. This assay detects only the potential pathogenic autoAbs against adult and fetal subtypes of the nAChR extracellular part (38). We found 18 sera, derived from 7 patients with MG, negative or ambiguous by CBA (Figure 1 and Table 1). This suggests that these patients with MG do not harbor autoAbs targeting extracellular parts of the nAChR or that these autoAbs could not be detected by this method, due to their low concentration in the serum. Interestingly, 3 out of these 7 patients, of whom the clinical data were available, belong to the I-IIA scale according to the MG Foundation of America (MGFA) clinical classification (Table 1).

### Depletion of AutoAbs Against the Extracellular Domain of the nAChR Subunits

To characterize the autoAbs' subunit-ECD specificity, depletion of autoAbs against the various nAChR ECDs from serum samples was achieved by immunoadsorption (Figure 1). For the following experiments, we used immunoadsorption protocols previously established in our laboratory (24, 49). Each immunoadsorption column contained sepharose beads with immobilized either one of the ECDs of the five nAChR subunits (α1, β1, γ, δ, or ε) or only BSA (23, 24, 49). To ensure that all the autoAbs incubated with the immobilized ECDs could be depleted, we used 0.125 pmoles antibodies (Abs)/mg of ECD-sepharose beads, since the capacity of the columns was determined in previous studies to be 1.5 pmoles Abs/mg of immobilized α1-ECD sepharose beads and 5 pmoles Abs/mg of β1-ECD sepharose beads (24, 49).

All the CBA+ MG patients' sera were incubated with the 6 proteins (α1-, β1-, γ-, δ-, ε-ECD, and BSA) immobilized on sepharose beads; unbound autoAbs were then quantified by RIPA and the percentage of immunoadsorption by each column was calculated (Figure 1 and Table 1). From the group of CBA− MG patients, only 3 out of the 7 patients were chosen to be tested by immunoadsorption to verify the absence of any extracellular autoAbs. As expected, there was practically no depletion of autoAbs after incubation with columns containing beads with immobilized nAChR ECDs (Figure 1 and Table 1), confirming the CBA result.

After immunoadsorption, the depleted sera from CBA+ MG patients were further qualified by CBA. More specifically, we investigated if all the autoAbs directed against extracellular epitopes of the nAChR were removed. Based on these experiments, we divided the MG patients tested into the three distinct groups, as given in Figure 1 and described below:

(a) Patients With MG Harboring autoAbs Against the α1 Subunit.

**Figure 2:**
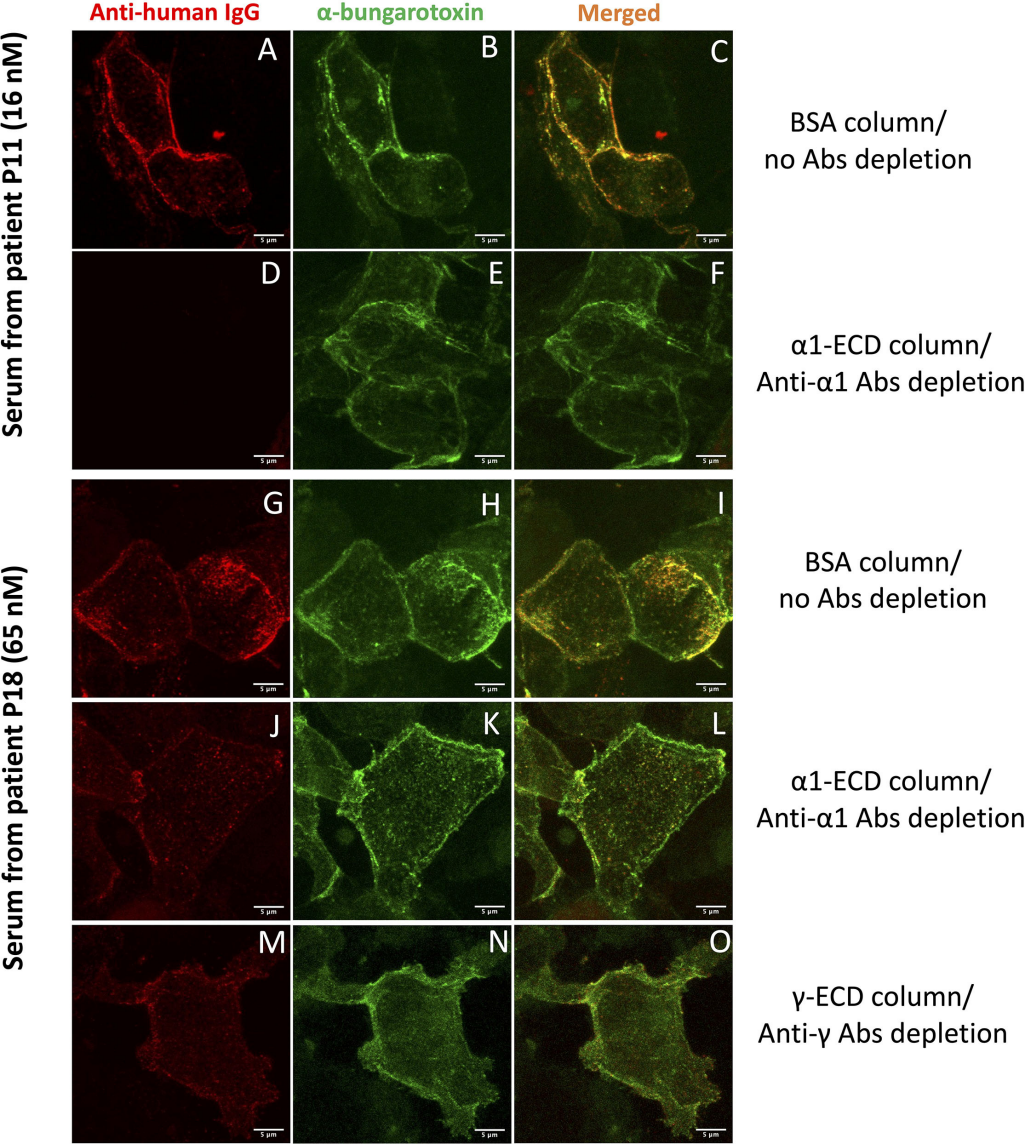
Cell-based assay (CBA) performed with sera after the immunoadsorption assay. HEK293T cells overexpressing a mixture of adult and fetal subtypes of the nAChR were used in the assay. **(A–F)** show cells incubated with serum derived from the patient P11 treated with BSA sepharose beads **(A–C)**, where no autoAbs were depleted or treated with α1-ECD sepharose beads **(D–F)**, where the anti-α1 autoAbs were depleted. **(G–O)** show cells incubated with serum derived from the patient P18 treated with BSA **(G–I)**, α1-ECD **(J–L)**, and γ-ECD **(M–O)** sepharose beads. The first column shows the binding of the specific anti-nAChR autoAbs contained in the serum, visualized by Alexa Fluor-555 labeled anti-human IgG Ab, the second column shows the total number of the nAChR on the cell surface of HEK293T stained with Alexa Fluor-488 labeled α-bungarotoxin. The third column shows merged confocal images of the anti-human IgG and α-bungarotoxin. Images were taken by Leica confocal TCS-SP8 microscope.

Five out of 13 CBA+ patients (P8-P12) had anti-α1 autoAbs (Figure 1 and Table 1). After immunoadsorption with the immobilized α1-ECD, these sera were found negative or ambiguous by CBA (Figures 2A–F), suggesting that the vast majority of autoAbs targeting extracellular epitopes were depleted by immunoadsorption. Also, data from P8 revealed that an increase of the anti-α1 autoAbs attributed to a higher MGFA score (Table 1).

(b) Patients With MG Harboring autoAbs Against the non-α1 Subunits.

Three out of 13 CBA+ patients had non-anti-α1 autoAbs (Figure 1 and Table 1); one patient had anti-γ autoAbs (P13) and two had anti-β1 autoAbs (P14, P15). After immunoadsorption, all sera of the P13 that had been incubated with the γ- immobilized ECD and all sera of the P14 and P15, incubated with the β1-immobilized ECD were negative or ambiguous by CBA. Moreover, P13 who harbors anti-γ autoAbs belongs to the MGFA-I clinical classification, indicating ocular MG (Table 1).

(c) Patients With MG Harboring autoAbs Against the α1 and non-α1 Subunits.

Five out of 13 CBA+ patients had anti-α1 and non-anti-α1 autoAbs. More specifically, P16 and P18 had anti-α1 and anti-γ autoAbs, P17 and P20 had anti-α1, anti-β1, and anti-γ autoAbs and P19 had anti-α1 and anti-β1 autoAbs (Figure 1 and Table 1). Interestingly, anti-β1 autoAbs were detected in P19 and P20 for the first time at the second and third samples, respectively, while anti-α1 autoAbs were not detected at those time points (Figure 1 and Table 1). The sera that were treated with the corresponding column when tested by CBA produced a reduced signal (Figures 2G–O). In some sera, the reduction of the signal in the CBA was higher than the percentage of immunoadsorption. This is probably due to the depletion of the autoAbs against extracellular epitopes of the receptor after immunoadsorption. The remaining autoAbs were still detected by RIPA but not CBA; this implies that they probably target intracellular epitopes. The clinical data for P16, P17, P19, and P20 indicate that the increase of the non-anti-α1 autoAbs correlates with a decrease in the MGFA score (Table 1).

## Discussion

Myasthenia gravis is an autoimmune disease caused mainly by autoAbs targeting the nAChR on the NMJ and results in the impairment of neuromuscular transmission and muscle weakness (4). Anti-nAChR autoAbs are heterogeneous and may target all the subunits of the receptor with demonstrated different potency for inducing experimental autoimmune MG in rodent models (26, 50, 51). Moreover, epitope spreading against intracellular epitopes, may occur at later stages of MG, as shown in the experimental autoimmune MG rat model, probably due to tissue damage (52).

Here, we studied the specificity of the anti-nAChR autoAbs in sera derived from 20 anti-nAChR positive patients with MG at different time points. We found that 7 out of the 20 patients with MG were negative or ambiguous by CBA (Figure 1 and Table 1). This suggests that the majority of the autoAbs found in these patients' sera is probably against the intracellular parts of the receptor, which are detectable only by RIPA. Interestingly, according to RIPA, these sera had high anti-nAChR autoAbs titer. On the other hand, one could claim that the negative signal of CBA could be attributed to a concentration of autoAbs against extracellular parts of the nAChR well-below the detection limits of the CBA. However, previous studies have also shown patients with MG to be positive by RIPA and negative by CBA (53). The majority of these patients did not have the clinical profile of a neuromuscular transmission disorder, implying that they had no pathogenic autoAbs, which probably recognized intracellular parts of the nAChR (53). Despite the absence of clinical data, we believe that 7 CBA− MG patients in this study fall into the same category as that other study.

Having detected CBA+ patients, we proceeded to further characterization of the autoAbs presented in their sera regarding their subunit specificity. We used immunoadsorption columns appropriate for the depletion of autoAbs targeting the nAChR subunits, as described previously (24, 49). In brief, the columns contained immobilized either the ECD of one of the five subunits of the nAChR (α1, β1, γ, δ, or ε) or BSA. Each serum was incubated with each column and the unbound autoAbs were quantified by RIPA. Moreover, to qualify the unbound autoAbs which recognize extracellular parts of the nAChR, we performed CBA. It is worth mentioning that by testing each serum for the presence of autoAbs against all the five subunits, we also tested the specificity of the bound autoAbs. Based on the results from both techniques, we concluded that CBA+ MG patients tested here can be divided into the three distinct groups: (a) patients with MG harboring anti-α1 autoAbs (5/13), (b) patients with MG harboring non-anti-α1 autoAbs (3/13), and (c) patients with MG harboring anti-α1 and non-anti-α1 autoAbs (5/13) (Figure 1 and Table 1). The CBA signal of all the sera after immunoadsorption was reduced in agreement with the immunoadsorption treatment (Figures 2A–F). In fact, in some sera, we observed a higher reduction in the CBA signal (Figures 2G–O), compared to the depletion of autoAbs detected in RIPA after immunoadsorption, e.g., although the immunoadsorption percentage of P8's serum after treatment with the immobilized α1-ECD was only 58%, the CBA performed after the immunoadsorption produced no signal. This is probably due to the depletion of the autoAbs against the extracellular epitopes of the receptor after the immunoadsorption. The fact that the remaining autoAbs were detected by RIPA but not by CBA implies that they target intracellular epitopes. In other sera, the CBA signal was negative or ambiguous, suggesting that most autoAbs against the extracellular part of the nAChR were depleted by immunoadsorption. In agreement with previous works, in none of the samples anti-δ or anti-ε autoAbs were detected (23, 24). In general, we observed that the increase of the non-anti-α1 autoAbs correlates with improvement in the disease manifestation (Table 1).

The pathogenicity of the anti-α1 autoAbs is well-characterized. The α1 subunit is immunodominant and it can induce experimental autoimmune MG in rats (51). Accordingly, in the anti-α1 autoAbs positive P8 patient, we observed that the increase of the anti-nAChR autoAbs titer correlates with disease deterioration (Table 1). The pathogenicity of the anti-β1 autoAbs is less studied and these are thought to be less pathogenic than the anti-α1 autoAbs (26). In fact, in P19 the MGFA score decreased when the autoAbs specificity switched from anti-α1 to anti-β1 autoAbs. Interestingly, in the P20 patient, despite the great increase of the anti-nAChR autoAbs titer, there was no change in the patient's clinical profile upon decrease of the anti-α1 autoAbs and increase of the anti-β1 autoAbs (Table 1). Although, the pathogenicity of the anti-γ autoAbs is proved in newborns, in adults they are less pathogenic and may recognize the fetal subtype of the nAChR presented on the extraocular muscle (29, 30, 54). Indeed, P13 who is positive for anti-γ autoAbs has a low MGFA score, which indicates ocular MG (Table 1). Moreover, the disease symptoms improve when the anti-γ autoAbs in P17 increase over the anti-α1 autoAbs. Also, there was no difference in the patient's clinical profile when the anti-nAChR autoAbs titer increased, probably due to the presence of only anti-γ autoAbs (Table 1). By these observations, we had previously reported a double positive MG patient (anti-nAChR and anti-MuSK autoAbs positive) who was presented with MuSK phenotype (25). This patient's clinical manifestation of the disease was not affected by the increase of the anti-nAChR autoAbs titer. After immunoadsorption, we showed that in all sera from different time points, the patient had relatively small amounts of anti-α1 autoAbs and the vast majority of autoAbs were directed against the β1 and γ subunits. We concluded that the patient did not show any clinical deterioration, because the pathogenic anti-α1 autoAbs were always in low concentration, while the increase of the anti-nAChR autoAbs titer was attributed to the increase of only the less pathogenic anti-β1 and anti-γ autoAbs (25).

Although we do not have a complete clinical profile of all patients, our results support the idea that additional analysis of the autoAbs of patients with MG can provide additional information to the clinicians about the patients' status. This study presents the importance of the CBA technique in the MG diagnosis. It seems that some anti-nAChR positive patients with MG do not harbor pathogenic autoAbs against the extracellular parts of the nAChR or their concentration is under the detection limit of CBA, something that may affect the decision of treatment's strategy. Moreover, we conclude that anti-nAChR positive MG patients can be divided into distinct groups, based on their autoAbs specificity. Consequently, we propose the combination of RIPA and CBA for the follow-up of the MG patients. The former is to be used for the quantification of the autoAbs and the latter for the identification of the fluctuation of the pathogenic ones.

In future studies, we aim to enlarge our sample group and continue the study of the anti-nAChR autoAbs specificity in MG patients. Moreover, we plan to collect more clinical data from patients with MG and investigate in-depth the correlation of the clinical presentation with autoAbs specificity.

## Data Availability Statement

The original contributions presented in the study are included in the article/supplementary material, further inquiries can be directed to the corresponding authors.

## Ethics Statement

The studies involving human participants were reviewed and approved by HPI Ethics Committee. The patients/participants provided their written informed consent to participate in this study.

## Author Contributions

MM conducted the CBAs and immunoadsorption experiments, wrote and edited the manuscript. VZ contributed to the concept of the study, provided the clinical data of the patients, and interpreted the data. MB performed the RIPA experiments. AH conducted confocal microscopy experiments and edited the manuscript. MZ expressed the nAChR ECDs and edited the manuscript. PZ designed the experiments, interpreted the data, and edited the manuscript. All the authors reviewed and approved the final version of the manuscript.

## Funding

This study was supported by the Stavros Niarchos Foundation (SNF) grant and institutional funding.

## Conflict of Interest

The authors declare that the research was conducted in the absence of any commercial or financial relationships that could be construed as a potential conflict of interest.

## Publisher's Note

All claims expressed in this article are solely those of the authors and do not necessarily represent those of their affiliated organizations, or those of the publisher, the editors and the reviewers. Any product that may be evaluated in this article, or claim that may be made by its manufacturer, is not guaranteed or endorsed by the publisher.

## References

[1] Gilhus NE Verschuuren JJ. Myasthenia gravis: Subgroup classification and therapeutic strategies. Lancet Neurol. (2015) 14:1023–36. 10.1016/S1474-4422(15)00145-3 26376969

[2] Gilhus NE Skeie GO Romi F Lazaridis K Zisimopoulou P Tzartos S. Myasthenia gravis - autoantibody characteristics and their implications for therapy. Nat Rev Neurol. (2016) 12:259–68. 10.1038/nrneurol.2016.44 27103470

[3] Wang S Breskovska I Gandhy S Punga AR Guptill JT Kaminski HJ. Advances in autoimmune myasthenia gravis management. Expert Rev Neurother. (2018) 18:573–88. 10.1080/14737175.2018.1491310 29932785 PMC6289049

[4] Hughes BW Moro De Casillas ML Kaminski HJ. Pathophysiology of myasthenia gravis. Semin Neurol. (2004) 24:21–30. 10.1055/s-2004-829585 15229789

[5] Lindstrom JM Seybold ME Lennon VA Whittingham S Duane DD. Antibody to acetylcholine receptor in myasthenia gravis. Prevalence, clinical correlates, and diagnostic value. Neurology. (1976) 26:1054–9. 10.1212/WNL.26.11.1054 988512

[6] Rodríguez Cruz PM Cossins J Beeson D Vincent A. The neuromuscular junction in health and disease: molecular mechanisms governing synaptic formation and homeostasis. Front Mol Neurosci. (2020) 13:610964. 10.3389/fnmol.2020.610964 33343299 PMC7744297

[7] Hoch W McConville J Helms S Newsom-Davis J Melms A Vincent A. Auto-antibodies to the receptor tyrosine kinase MuSK in patients with myasthenia gravis without acetylcholine receptor antibodies. Nat Med. (2001) 7:365–8. 10.1038/85520 11231638

[8] Higuchi O Hamuro J Motomura M Yamanashi Y. Autoantibodies to low-density lipoprotein receptor-related protein 4 in myasthenia gravis. Ann Neurol. (2011) 69:418–22. 10.1002/ana.22312 21387385

[9] Zhang B Tzartos JS Belimezi M Ragheb S Bealmear B Lewis RA. Autoantibodies to lipoprotein-related protein 4 in patients with double-seronegative myasthenia gravis. Arch Neurol. (2012) 69:445–51. 10.1001/archneurol.2011.2393 22158716

[10] Pevzner A Schoser B Peters K Cosma N-C Karakatsani A Schalke B. Anti-LRP4 autoantibodies in AChR- and MuSK-antibody-negative myasthenia gravis. J Neurol. (2012) 259:427–35. 10.1007/s00415-011-6194-7 21814823

[11] Gilhus NE Tzartos S Evoli A Palace J Burns TM Verschuuren JJGM. Myasthenia gravis. Nat Rev Dis Prim. (2019) 5:50. 10.1038/s41572-019-0079-y 31048702

[12] Gallardo E Martínez-Hernández E Titulaer MJ Huijbers MG Martínez MA Ramos A. Cortactin autoantibodies in myasthenia gravis. Autoimmun Rev. (2014) 13:1003–7. 10.1016/j.autrev.2014.08.039 25193850

[13] Zhang B Shen C Bealmear B Ragheb S Xiong W-C Lewis RA. Autoantibodies to agrin in myasthenia gravis patients. PLoS ONE. (2014) 9:e91816. 10.1371/journal.pone.0091816 24632822 PMC3954737

[14] Gasperi C Melms A Schoser B Zhang Y Meltoranta J Risson V. Anti-agrin autoantibodies in myasthenia gravis. Neurology. (2014) 82:1976–83. 10.1212/WNL.0000000000000478 24793185

[15] Skeie GO Mygland Å Treves S Gilhus NE Aarli JA Zorzato F. Ryanodine receptor antibodies in myasthenia gravis: epitope mapping and effect on calcium release *in vitro*. Muscle Nerve. (2003) 27:81–9. 10.1002/mus.10294 12508299

[16] Aarli JA Stefansson K Marton LSG Wollmann RL. Patients with myasthenia gravis and thymoma have in their sera IgG autoantibodies against titin. Clin Exp Immunol. (1990) 82:284–8. 10.1111/j.1365-2249.1990.tb05440.x 2242609 PMC1535140

[17] Albuquerque EX Pereira EFR Alkondon M Rogers SW. Mammalian nicotinic acetylcholine receptors: from structure to function. Physiol Rev. (2009) 89:73–120. 10.1152/physrev.00015.2008 19126755 PMC2713585

[18] Karlin A. Ion channel structure: emerging structure of the Nicotinic Acetylcholine receptors. Nat Rev Neurosci. (2002) 3:102–14. 10.1038/nrn731 11836518

[19] Le Novère N Corringer P-J Changeux J-P. The diversity of subunit composition in nAChRs: evolutionary origins, physiologic and pharmacologic consequences. J Neurobiol. (2002) 53:447–56. 10.1002/neu.10153 12436412

[20] Unwin N. Refined structure of the nicotinic acetylcholine receptor at 4A resolution. J Mol Biol. (2005) 346:967–89. 10.1016/j.jmb.2004.12.031 15701510

[21] Tzartos S Hochschwender S Vasquez P Lindstrom J. Passive transfer of experimental autoimmune myasthenia gravis by monoclonal antibodies to the main immunogenic region of the acetylcholine receptor. J Neuroimmunol. (1987) 15:185–94. 10.1016/0165-5728(87)90092-0 3495549

[22] Tzartos SJ Lindstrom JM. Monoclonal antibodies used to probe acetylcholine receptor structure: localization of the main immunogenic region and detection of similarities between subunits. Proc Natl Acad Sci USA. (1980) 77:755–9. 10.1073/pnas.77.2.755 6153804 PMC348359

[23] Zisimopoulou P Lagoumintzis G Poulas K Tzartos SJ. Antigen-specific apheresis of human anti-acetylcholine receptor autoantibodies from myasthenia gravis patients' sera using *Escherichia coli*-expressed receptor domains. J Neuroimmunol. (2008) 200:133–41. 10.1016/j.jneuroim.2008.06.002 18603305

[24] Kostelidou K Trakas N Tzartos SJ. Extracellular domains of the β, γ and ε subunits of the human acetylcholine receptor as immunoadsorbents for myasthenic autoantibodies: a combination of immunoadsorbents results in increased efficiency. J Neuroimmunol. (2007) 190:44–52. 10.1016/j.jneuroim.2007.07.018 17764755

[25] Zouvelou V Michail M Belimezi M Zisimopoulou P. Subunit specificity of the acetylcholine receptor antibodies in double seropositive myasthenia gravis. Muscle Nerve. (2021) 63:E36–7. 10.1002/mus.27177 33471417

[26] Kordas G Lagoumintzis G Sideris S Poulas K Tzartos SJ. Direct proof of the *in vivo* pathogenic role of the AChR autoantibodies from myasthenia gravis patients. PLoS ONE. (2014) 9:0108327. 10.1371/journal.pone.0108327 25259739 PMC4178151

[27] Vincent A Newland C Beeson D Riemersma S Newsom-Davis J Brueton L. Arthrogryposis multiplex congenita with maternal autoantibodies specific for a fetal antigen. Lancet. (1995) 346:24–5. 10.1016/S0140-6736(95)92652-6 7603140

[28] Kaminski HJ Kusner LL Block CH. Expression of acetylcholine receptor isoforms at extraocular muscle endplates. Invest Ophthalmol Vis Sci. (1996) 37:345–51. 8603839

[29] Shi QG Wang ZH Ma XW Zhang DQ Yang CS Shi FD. Clinical significance of detection of antibodies to fetal and adult acetylcholine receptors in myasthenia gravis. Neurosci Bull. (2012) 28:469–74. 10.1007/s12264-012-1256-0 22961471 PMC3600950

[30] MacLennan C Beeson D Buijs AM Vincent A Newsom-Davis J. Acetylcholine receptor expression in human extraocular muscles and their susceptibility to myasthenia gravis. Ann Neurol. (1997) 41:423–31. 10.1002/ana.410410404 9124798

[31] Vincent A Newsom-Davis J. Acetylcholine receptor antibody as a diagnostic test for myasthenia gravis: results in 153 validated cases and 2967 diagnostic assays. J Neurol Neurosurg Psychiatry. (1985) 48:1246–52. 10.1136/jnnp.48.12.1246 4087000 PMC1028609

[32] Yang L Maxwell S Leite MI Waters P Clover L Fan X. Non-radioactive serological diagnosis of myasthenia gravis and clinical features of patients from Tianjin, China. J Neurol Sci. (2011) 301:71–6. 10.1016/j.jns.2010.10.023 21131008

[33] Hewer R Matthews I Chen S McGrath V Evans M Roberts E. A sensitive non-isotopic assay for acetylcholine receptor autoantibodies. Clin Chim acta. (2006) 364:159–66. 10.1016/j.cccn.2005.05.035 16051208

[34] Furukawa S Akazawa S Furukawa Y Kamo I Satoyoshi E Hayashi K. A practical enzyme immunoassay for anti-acetylcholine receptor antibodies in myasthenia gravis. J Neuroimmunol. (1984) 6:397–409. 10.1016/0165-5728(84)90065-1 6384263

[35] Leite MI Jacob S Viegas S Cossins J Clover L Morgan BP. IgG1 antibodies to acetylcholine receptors in “seronegative” myasthenia gravis. Brain. (2008) 131:1940–52. 10.1093/brain/awn092 18515870 PMC2442426

[36] Gastaldi M Scaranzin S Businaro P Mobilia E Benedetti L Pesce G. Improving laboratory diagnostics in myasthenia gravis. Expert Rev Mol Diagn. (2021) 21:579–90. 10.1080/14737159.2021.1927715 33970749

[37] Rodriguez Cruz PM Huda S López-Ruiz P Vincent A. Use of cell-based assays in myasthenia gravis and other antibody-mediated diseases. Exp Neurol. (2015) 270:66–71. 10.1016/j.expneurol.2015.01.011 25783660

[38] Vincent A Waters P Leite MI Jacobson L Koneczny I Cossins J. Antibodies identified by cell-based assays in myasthenia gravis and associated diseases. Ann N Y Acad Sci. (2012) 1274:92–8. 10.1111/j.1749-6632.2012.06789.x 23252902

[39] Rodríguez Cruz PM Al-Hajjar M Huda S Jacobson L Woodhall M Jayawant S. Clinical features and diagnostic usefulness of antibodies to clustered acetylcholine receptors in the diagnosis of Seronegative Myasthenia Gravis. JAMA Neurol. (2015) 72:642–9. 10.1001/jamaneurol.2015.0203 25894002 PMC6044422

[40] Yan C Zhao R Song J Feng X Xi J Luo S. Comparison of anti-acetylcholine receptor profiles between Chinese cases of adult- and juvenile-onset myasthenia gravis using cell-based assays. J Neuroimmunol. (2020) 349:577403. 10.1016/j.jneuroim.2020.577403 32992216

[41] Jacob S Viegas S Leite MI Webster R Cossins J Kennett R. Presence and pathogenic relevance of antibodies to clustered acetylcholine receptor in ocular and generalized myasthenia gravis. Arch Neurol. (2012) 69:994–1001. 10.1001/archneurol.2012.437 22689047

[42] Hong Y Zisimopoulou P Trakas N Karagiorgou K Stergiou C Skeie GO. Multiple antibody detection in “seronegative” myasthenia gravis patients. Eur J Neurol. (2017) 24:844–50. 10.1111/ene.13300 28470860

[43] Park KH Waters P Woodhall M Lang B Smith T Sung J-J. Myasthenia gravis seronegative for acetylcholine receptor antibodies in South Korea: autoantibody profiles and clinical features. PLoS ONE. (2018) 13:e0193723. 10.1371/journal.pone.0193723 29518096 PMC5843234

[44] Yan C Li W Song J Feng X Xi J Lu J. Cell-based versus enzyme-linked immunosorbent assay for the detection of acetylcholine receptor antibodies in Chinese Juvenile Myasthenia Gravis. Pediatr Neurol. (2019) 98:74–9. 10.1016/j.pediatrneurol.2019.01.016 31307830

[45] Cai Y Han L Zhu D Peng J Li J Ding J. A stable cell line expressing clustered AChR: a novel cell-based assay for Anti-AChR antibody detection in Myasthenia Gravis. Front Immunol. (2021) 12:666046. 10.3389/fimmu.2021.666046 34305897 PMC8297518

[46] Zhao G Wang X Yu X Zhang X Guan Y Jiang J. Clinical application of clustered-AChR for the detection of SNMG. Sci Rep. (2015) 5:10193. 10.1038/srep10193 26068604 PMC4464178

[47] Psaridi-Linardaki L Mamalaki A Remoundos M Tzartos SJ. Expression of soluble ligand- and antibody-binding extracellular domain of human muscle acetylcholine receptor alpha subunit in yeast Pichia pastoris. Role of glycosylation in alpha-bungarotoxin binding. J Biol Chem. (2002) 277:26980–6. 10.1074/jbc.M110731200 12015305

[48] Kostelidou K Trakas N Zouridakis M Bitzopoulou K Sotiriadis A Gavra I. Expression and characterization of soluble forms of the extracellular domains of the β, γ and ε subunits of the human muscle acetylcholine receptor. FEBS J. (2006) 273:3557–68. 10.1111/j.1742-4658.2006.05363.x 16884496

[49] Psaridi-Linardaki L Trakas N Mamalaki A Tzartos SJ. Specific immunoadsorption of the autoantibodies from myasthenic patients using the extracellular domain of the human muscle acetylcholine receptor α-subunit. Development of an antigen-specific therapeutic strategy. J Neuroimmunol. (2005) 159:183–91. 10.1016/j.jneuroim.2004.10.002 15652418

[50] Tzartos SJ Bitzopoulou K Gavra I Kordas G Jacobson L Kostelidou K. Antigen-specific apheresis of pathogenic autoantibodies from myasthenia gravis sera. Ann N Y Acad Sci. (2008) 1132:291–9. 10.1196/annals.1405.017 18567880

[51] Lazaridis K Baltatzidi V Trakas N Koutroumpi E Karandreas N Tzartos SJ. Characterization of a reproducible rat EAMG model induced with various human acetylcholine receptor domains. J Neuroimmunol. (2017) 303:13–21. 10.1016/j.jneuroim.2016.12.011 28038891

[52] Feferman T Im S-H Fuchs S Souroujon MC. Breakage of tolerance to hidden cytoplasmic epitopes of the acetylcholine receptor in experimental autoimmune myasthenia gravis. J Neuroimmunol. (2003) 140:153–8. 10.1016/S0165-5728(03)00209-1 12864983

[53] Maddison P Sadalage G Ambrose PA Jacob S Vincent A. False-positive acetylcholine receptor antibody results in patients without myasthenia gravis. J Neuroimmunol. (2019) 332:69–72. 10.1016/j.jneuroim.2019.04.001 30959340

[54] Dalton P Clover L Wallerstein R Stewart H Genzel-Boroviczeny O Dean A. Fetal arthrogryposis and maternal serum antibodies. Neuromuscul Disord. (2006) 16:481–91. 10.1016/j.nmd.2006.05.015 16919948

